# Impact of intensified training and carbohydrate supplementation on immunity and markers of overreaching in highly trained cyclists

**DOI:** 10.1007/s00421-016-3340-z

**Published:** 2016-02-23

**Authors:** Ida S. Svendsen, Sophie C. Killer, James M. Carter, Rebecca K. Randell, Asker E. Jeukendrup, Michael Gleeson

**Affiliations:** School of Sport, Exercise and Health Sciences, Loughborough University, Leicestershire, LE11 3TU UK; Gatorade Sports Science Institute, PepsiCo Global Nutrition R&D, Beaumont Park, Leicester, UK

**Keywords:** Overreaching, Cytokine, Lymphocyte, Interleukin, Cortisol, ACTH

## Abstract

**Purpose:**

To determine effects of intensified training (IT) and carbohydrate supplementation on overreaching and immunity.

**Methods:**

In a randomized, double-blind, crossover design, 13 male cyclists (age 25 ± 6 years, $\dot{V}O_{2\hbox{max} }$ 72 ± 5 ml/kg/min) completed two 8-day periods of IT. On one occasion, participants ingested 2 % carbohydrate (L-CHO) beverages before, during and after training sessions. On the second occasion, 6 % carbohydrate (H-CHO) solutions were ingested before, during and after training, with the addition of 20 g of protein in the post-exercise beverage. Blood samples were collected before and immediately after incremental exercise to fatigue on days 1 and 9.

**Results:**

In both trials, IT resulted in decreased peak power (375 ± 37 vs. 391 ± 37 W, *P* < 0.001), maximal heart rate (179 ± 8 vs. 190 ± 10 bpm, *P* < 0.001) and haematocrit (39 ± 2 vs. 42 ± 2 %, *P* < 0.001), and increased plasma volume (*P* < 0.001). Resting plasma cortisol increased while plasma ACTH decreased following IT (*P* < 0.05), with no between-trial differences. Following IT, antigen-stimulated whole blood culture production of IL-1α was higher in L-CHO than H-CHO (0.70 (95 % CI 0.52–0.95) pg/ml versus 0.33 (0.24–0.45) pg/ml, *P* < 0.01), as was production of IL-1β (9.3 (95 % CI 7–10.4) pg/ml versus 6.0 (5.0–7.8) pg/ml, *P* < 0.05). Circulating total leukocytes (*P* < 0.05) and neutrophils (*P* < 0.01) at rest increased following IT, as did neutrophil:lymphocyte ratio and percentage CD4+ lymphocytes (*P* < 0.05), with no between-trial differences.

**Conclusion:**

IT resulted in symptoms consistent with overreaching, although immunological changes were modest. Higher carbohydrate intake was not able to alleviate physiological/immunological disturbances.

## Introduction

Overload is a key training principle used by athletes and coaches to improve physical performance. Periods of intensified training (IT) are therefore commonly incorporated over the course of a training season. However, when high training load is combined with insufficient recovery and/or other stressors such as lack of sleep, psychological stress, environmental stress or inadequate energy intake, it can lead to a state of overreaching and place athletes at risk of developing symptoms of overtraining syndrome (Meeusen et al. 2013). The primary marker for the diagnosis of overreaching or overtraining syndrome is a reduction in sport-specific performance. Functional overreaching results in acute fatigue and a short-term decrement in performance lasting a few days, typically followed by a subsequent supercompensation. Non-functional overreaching or overtraining, on the other hand, is associated with long-term performance decrements that can last from several weeks to months, or even years (Meeusen et al. 2013).

Upper respiratory infections are the most common medical complaints affecting athletes (Fricker et al. 2005; Reeser et al. 2003), with the occurrence being particularly high during times of intense training or competition (Nieman 2000). Much anecdotal evidence also indicates increased upper respiratory infection incidence in athletes with symptoms of overtraining syndrome. This is not altogether surprising, as previous studies have reported reduced salivary secretory immunoglobulin-A (Gleeson et al. 1995; Mackinnon and Hooper 1994), reduced numbers of natural killer cells (Gleeson et al. 1995; Meyer et al. 2004) and CD3+ and CD4+ cells (Baj et al. 1994), impaired neutrophil function (Baj et al. 1994; Robson-Ansley et al. 2007), reduced glutamine/glutamate ratio (Coutts et al. 2007; Halson et al. 2003; Parrybillings et al. 1992; Rowbottom et al. 1995) and elevated plasma concentrations of interleukin(IL)-6 and creatine kinase at rest (Robson-Ansley et al. 2007) in response to IT. In addition, studies suggest that the capacity of immune cells to produce cytokines in response to an immune challenge may be impaired (Baj et al. 1994; Lancaster et al. 2004; Morgado et al. 2012). Cytokines play a key, pleiotropic role in orchestrating the responses of lymphocytes, macrophages and other immune cells during an infection (Biron et al. 1999; Samuel 2001; Trinchieri 1997). Disturbances to the profile of cytokine production in response to IT may therefore increase infection susceptibility. Gleeson et al. (2011a, b) found that athletes with a high training load (≥11 h moderate–high-intensity training per week) had more than twice as many upper respiratory tract infections (URTI) episodes, alongside approximately threefold higher resting IL-2, IL-4 and IL-10 production in response to antigen challenge compared to athletes with a low training volume (<6 h per week). An elevated anti-inflammatory response, and specifically increased IL-10 production, appears to be a risk factor for URTI in athletes (Gleeson et al. 2012). IL-10 is primarily produced by lymphocytes and, in particular, regulatory T (Treg) cells (Gleeson et al. 2011b); circulating numbers of Treg cells appear to be higher in physically active compared with sedentary individuals (Handzlik et al. 2013), but no studies to date have investigated changes in this cell population during IT.

Carbohydrate intake may play a role in the prevention of overreaching and alleviate associated immune disturbances (Achten et al. 2004; Halson et al. 2004; Costa et al. 2005), possibly via attenuation of the stress hormone response to exercise (Costa et al. 2005). Most competitive endurance athletes typically ingest some carbohydrate during prolonged bouts of training or competition. However, it is not clear whether increasing carbohydrate ingestion before, during and after exercise in line with American College of Sports Medicine (ACSM) guidelines (Rodriguez et al. 2009) can provide additional protection against overreaching and immune disturbances compared to more modest carbohydrate intakes in and around the exercise occasion.

The aim of the current study was therefore to determine the effects of 8 days of intensified cycling training with a self-selected diet supplemented with either low or high carbohydrate drinks before, during and after training sessions, on immunity and markers of overreaching in highly trained cyclists.

## Methods

### Subjects

Thirteen highly trained male cyclists provided their written, informed consent to participate in the study, which was approved by the Loughborough University ethical advisory committee. Prior to the start of the study, participants completed a health screening questionnaire. Subjects could be included if they were between 18 and 35 years of age, had a maximal oxygen uptake ($\dot{V}O_{2\hbox{max} }$) of ≥64 mL/kg/min, had been actively competing in cycling for at least 2 years and were currently engaging in a minimum of 6 h of cycling training per week. Participants were also required to be currently healthy and without upper respiratory symptoms or use of any medication during the past 4 weeks. Exclusion criteria were: smoking, suffering from, or with a history of, cardiac, hepatic, pulmonary, renal, neurological, haematological, psychiatric or gastrointestinal illness, or baseline haematological values (leukocyte and erythrocyte counts) outside the normal range. Baseline characteristics were as follows (mean ± SD): age 25 ± 6 years, body mass 69.7 ± 6.3 kg, height 1.78 ± 0.03 m, body fat percentage 13.4 ± 4.1 %, and $\dot{V}O_{2\hbox{max} }$ 72.2 ± 4.9 mL/kg/min, 5.0 ± 0.5 L/min.

### Study design

**Fig. 1:**
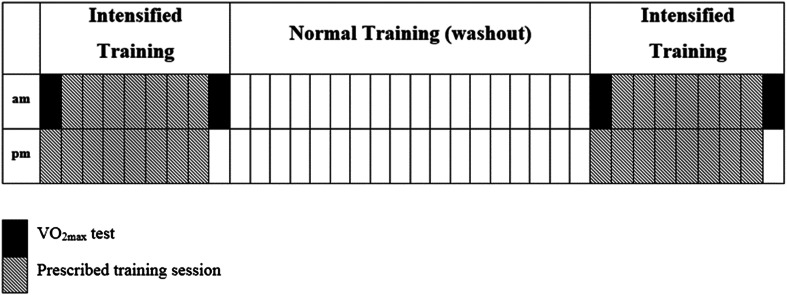
Diagrammatic representation of the intensified training protocol

The study was conducted using a double-blind, placebo-controlled, crossover design. Each participant completed two 8-day periods of intensified training in a randomized and counterbalanced order, separated by a 15-day washout period (Fig. 1). During one trial, participants were provided with high carbohydrate solutions to drink before, during and after every training session (H-CHO). In the other trial they were given a taste-matched low carbohydrate solution (L-CHO).

### Baseline training

Prior to the start of the study, participants were asked to record all of their training for a minimum of 2 weeks. Session structure and duration, type of exercise, heart rate and power output data were uploaded to an online training diary (TrainingPeaks, Peaksware LLC, Colorado). These data were analysed to give a baseline total weekly number of hours of cycling and training volume at different intensities and used to calculate and prescribe individual training plans for each IT period.

### Intensified training

During each 8-day IT period, participants were prescribed an individualized training plan with targets for training time, heart rate and power output set for every session. Heart rate and power targets were based on results from the initial $\dot{V}O_{2\hbox{max} }$ test. These were adjusted accordingly for the second trial if performance in the $\dot{V}O_{2\hbox{max} }$ test on day 1 differed significantly from that in the first trial. During each IT period, total training volume was doubled compared to baseline training, and time spent at high intensity more than trebled, with high-intensity training (Table 1, Zones 3+) comprising ~35 % of the total training time. Training was categorized into five heart rate zones.

**Table 1:** Heart rate zones used for categorization of training intensity

Low intensity	
Zone 1	55–71 % HR_max_
Zone 2	72–81 % HR_max_
High intensity	
Zone 3	82–87 % HR_max_
Zone 4	88–92 % HR_max_
Zone 5	>92 % HR_max_

All participants were prescribed two sessions per day, with every session including a high-intensity interval training component (Zones 3–5). In addition to maximal incremental tests, participants attended the laboratory for supervised training sessions on days 1, 2, 5, 6 and 8 of both trials.

All training was performed either out on the road or in the laboratory on the participant’s own bike equipped with a power meter (SRM PowerMeter Shimano DA7950 compact, SRM International, Jülich, Germany) and mounted on a stationary turbo trainer to provide resistance. Heart rate and power output were recorded continuously for every session with a sampling frequency of 4 Hz and stored on the SRM PowerControl 7. All training data were subsequently uploaded to and analysed in an online training diary (TrainingPeaks, Peaksware LLC, Colorado). Participants were required to complete every session prescribed in their training plan to be included in the study. The two IT periods were separated by a 15-day washout, during which participants were encouraged to take time to recover before returning to normal training. Participants were asked to continue recording and uploading training data during the washout period.

### Nutritional intervention

For both trials, participants were provided with a specified volume and composition of drink before, during and after every training session (Table 2). Drinks ingested before and during exercise were taste matched, and participants were informed that the only difference in the nutritional intervention between the two trials was that in one they would be provided with a recovery drink, whereas during the other the recovery drink would be replaced with a ‘recovery capsule’ (containing only cellulose).

**Table 2:** Volumes and compositions of drinks provided before, during and after training sessions in H-CHO and L-CHO trials

	H-CHO	L-CHO
Before exercise	118 mL 20 % CHO solution (24 g)	118 mL 2 % CHO solution (2.4 g)
During exercise	1 L per hour 6 % CHO solution (60 g/h)	1 L per hour 2 % CHO solution (20 g/h)
After exercise	1 × 500 mL 6 % CHO solution (30 g) 1 × 500 mL 2.7 % CHO/3.3 % PRO solution (13.5 g)	1 × 500 mL 2 % CHO solution (10 g) 1 × cellulose capsule (0 g)

Values in parentheses indicate carbohydrate content in grams

*CHO* carbohydrate, *PRO* protein

For a typical 2 h training session, this provided a total carbohydrate intake of 187.5 g in the H-CHO compared to 52.4 g in L-CHO (an additional 17 g (~0.25 g/kg) protein was provided after exercise in the H-CHO group, with no protein received in the L-CHO group). The composition of the drinks provided in the H-CHO trial was chosen to conform with the current ACSM guidelines on recommended carbohydrate intake for endurance exercise (Rodriguez et al. 2009). Participants were asked to refrain from ingesting any other sports nutrition products or nutritional supplements for the duration of the study and were provided with a diet diary in which to record weighed food and fluid intake for 3 days prior to and throughout the course of each trial. Dietary analyses were performed using CompEat Pro software (Nutrition Systems, Banbury, England).

### Maximal incremental test

On days 1 and 9 of each trial, subjects completed a continuous, incremental exercise test to volitional exhaustion on a magnetically braked cycle ergometer (IndoorTrainer, SRM International, Jülich, Germany) for determination of $\dot{V}O_{2\hbox{max} }$. Subjects arrived at the laboratory at 7:00 am following an overnight fast of at least 10 h and having abstained from caffeine for a minimum of 12 h. All tests were completed at the same time of the day to reduce inter- and intra-trial effects of diurnal variations in cytokine production and plasma cortisol. The test began at 60 W with increments of 35 W every 3 min. Rating of perceived exertion (RPE) was noted and heart rate measured continuously using short-range telemetry (Polar, Kempele, Finland). The expired gas was collected and analysed continuously using a computerized metabolic system with a mixing chamber (Moxus, AEI Technologies, Pennsylvania, USA) for determination of $\dot{V}E$, $\dot{V}O_{2}$ and $\dot{V}CO_{2}$. Using these data, fat and carbohydrate oxidation rates were calculated using stoichiometric equations, assuming that protein oxidation throughout the test was negligible (Brouwer 1957). The gas analysers were calibrated with certified calibration gases of known concentrations before every test. The flow turbine was calibrated before every test with a Micro Medical SM2125 series 3 L calibration syringe (CareFusion, Hoechberg, Germany). The same metabolic system with identical calibration routines was used for all performance tests. Subjects were asked to record and replicate as closely as possible their dietary intake during the 24 h leading up to each incremental test and were required to abstain from alcohol consumption 24 h previously.

### Blood sampling

On days 1 and 9 of each trial, blood samples were collected at rest immediately before the start of the maximal exercise test (PRE) and immediately after cessation of exercise (POST). On each occasion, a venous blood sample (11 mL) was obtained by venipuncture from an antecubital vein. Blood was collected into two vacutainer tubes (Becton–Dickinson, Oxford, UK) containing either lithium heparin or K_3_EDTA as anticoagulant. Haematological analysis was immediately performed on the K_3_EDTA sample, as outlined below.

### Blood cell counts

Blood samples in the K_3_EDTA vacutainer (4 mL) were used for determination of red blood cell concentration, haematocrit, haemoglobin and total and differential leukocyte counts using an automated cell counter (Ac.TTM^5^diff haematology analyser, Beckman Coulter, High Wycombe, UK). The coefficient of variation for all measured variables in our laboratory was <2.0 %. Samples were analysed in duplicate and the average of both values entered into analyses. Haematocrit and haemoglobin values were used to calculate changes in plasma volume using the equations of Dill and Costill (1974).

### Plasma cortisol and ACTH

The remaining K_3_EDTA blood sample was centrifuged for 10 min at 1500*g* and 4 °C. Plasma was aliquoted and stored at −20 °C until analysis. Plasma concentrations of cortisol were determined using a commercially available solid phase competitive enzyme-linked immunosorbent assay (IBL International, Hamburg, Germany), with analytical sensitivity of 2.46 ng/mL and intra-assay coefficient of variation of <3.0 %. Plasma concentrations of ACTH were determined using a commercially available two-site enzyme-linked immunosorbent assay (Biomerica, California), with analytical sensitivity of 0.22 pg/mL and intra-assay coefficient of variation of <6.0 %. Plates were read on a microtitre plate reader at 450 nm. All samples were assayed in duplicate.

### Antigen-stimulated cytokine production

Stimulated whole blood culture production of IFN-γ, TNF-α, IL-1α, IL-1β, IL-2, IL-4, IL-6, IL-8 and IL-10 was determined as follows: for each sample, 0.25 mL heparinized whole blood was added to 0.75 mL RPMI medium (Sigma Chemicals, Poole, UK) with no added stimulant or stimulant at a dilution of 1:2000. The stimulant used was a commercially available multi-antigen vaccine containing diphtheria, tetanus, acellular pertussis, poliomyelitis and *Haemophilus influenzae* type b antigens (Pediacel vaccine, Sanofi Pasteur, Maidenhead, UK). The whole blood culture was then incubated for 24 h at 37 °C and 5 % CO_2_. Following centrifugation for 4 min at 13,000*g* in a microcentrifuge, the supernatants were collected and stored frozen at −20 °C until analysis. Cytokine concentrations were determined with an Evidence Investigator System using the cytokine biochip array EV 3623 (Randox, County Antrim, UK). The intra-assay coefficient of variation was <10 % for all measured cytokines. The unstimulated values were then subtracted from stimulated values to isolate cytokine production specifically in response to the antigen stimulation and account for plasma concentrations and spontaneous production during the culture period.

### Lymphocyte subsets

Fluorescent-conjugated monoclonal antibodies were used to identify specific cell surface markers (CD4, CD25, CD127) via four colour flow cytometry (FACS-Calibur, Becton–Dickinson, Oxford, UK) with CellQuest analysis software (Becton–Dickinson Biosciences, Oxford, UK). Briefly, 10 μL of human regulatory T cell cocktail (Becton–Dickinson Biosciences, Oxford, UK) was added to 120 μL heparinized whole blood and incubated in the dark for 20 min on ice. Erythrocytes were then lysed by adding 1.5 mL of lysis solution (FACS lysis buffer, Becton–Dickinson Biosciences). The sample was subsequently incubated for a further 10 min, before centrifugation at 1500*g* and 4 °C for 6 min. The supernatant was aspirated and the cells re-suspended in phosphate-buffered saline solution (PBS) containing 0.1 % bovine serum albumin and 2 mM EDTA. The mixture was centrifuged for a further 6 min at 1600*g*, the supernatant aspirated and the cells re-suspended in 400 μL PBS. Forward-scatter versus side-scatter plots were used to gate lymphocytes based on size and density, with 50,000 lymphocyte events acquired per analysis. CD4+ lymphocytes were further gated to identify CD25+ cells, and those that were also CD127^low/−^. This was based on the finding that CD127 expression inversely correlated with FoxP3 (Liu et al. 2006) and can therefore be used to identify Treg cells. Values obtained represented a percentage of total lymphocytes for CD4+ CD25+ CD127^low/−^(Treg cells). Absolute values for CD4 + CD25 + CD127^low/−^ cells were calculated based on the total lymphocyte count obtained previously via the automated cell counter. This process was also repeated to determine CD4+ and CD4+ CD25+ cell counts.

### Statistical analysis

Data are presented as mean ± standard deviation (SD) unless otherwise stated. All statistical analyses were performed using IBM SPSS Statistics 22.0. The Shapiro–Wilk test was used to determine whether data were normally distributed. A two-way repeated measures analysis of variance was used to determine whether there were significant differences between L-CHO and H-CHO trials before and after intensified training. A post hoc test with Bonferroni correction for multiple comparisons was used to determine the location of variance. Variables found to be significantly non-normal were log transformed prior to analysis and are presented as back-transformed geometric mean with 95 % confidence interval (CI). Based on the results of Mauchly’s sphericity test, Greenhouse–Geisser corrections were applied for epsilon <0.75 and Huynd–Feldt corrections for epsilon >0.75 where violations of sphericity were identified. Statistical significance was accepted at the *P* < 0.05 level.

## Results

During the 8-day IT period, total training volume was increased from 9.3 ± 2.4 h/week at baseline, to 23.5 ± 3.4 h/week, with high-intensity training (Zones 3–5) increased from 2.6 ± 2.5 to 6.5 ± 4.0 h/week, with no differences between trials. During the 15-day washout period between the two trials, participants returned to their baseline level of training. Over the 8-day IT period, participants completed 24.3 ± 3.3 MJ of cycling, with no significant difference between the trials. Total daily energy intake was significantly lower in L-CHO compared to H-CHO (14.7 ± 2.7 vs. 17.4 ± 3.2 MJ/d, *P* < 0.001). This difference was primarily due to lower carbohydrate intake in L-CHO (505 ± 107 vs. 679 ± 105 g/d, *P* < 0.001; 7.2 ± 1.6 vs. 9.7 ± 1.5 g/kg/d, *P* < 0.05). However, in both groups, participants remained relatively weight stable, with a mean body mass change of +0.17 ± 1.14 kg in H-CHO and −0.55 ± 0.92 kg in L-CHO, with no significant difference between the two conditions (*P* = 0.144).

**Fig. 2:**
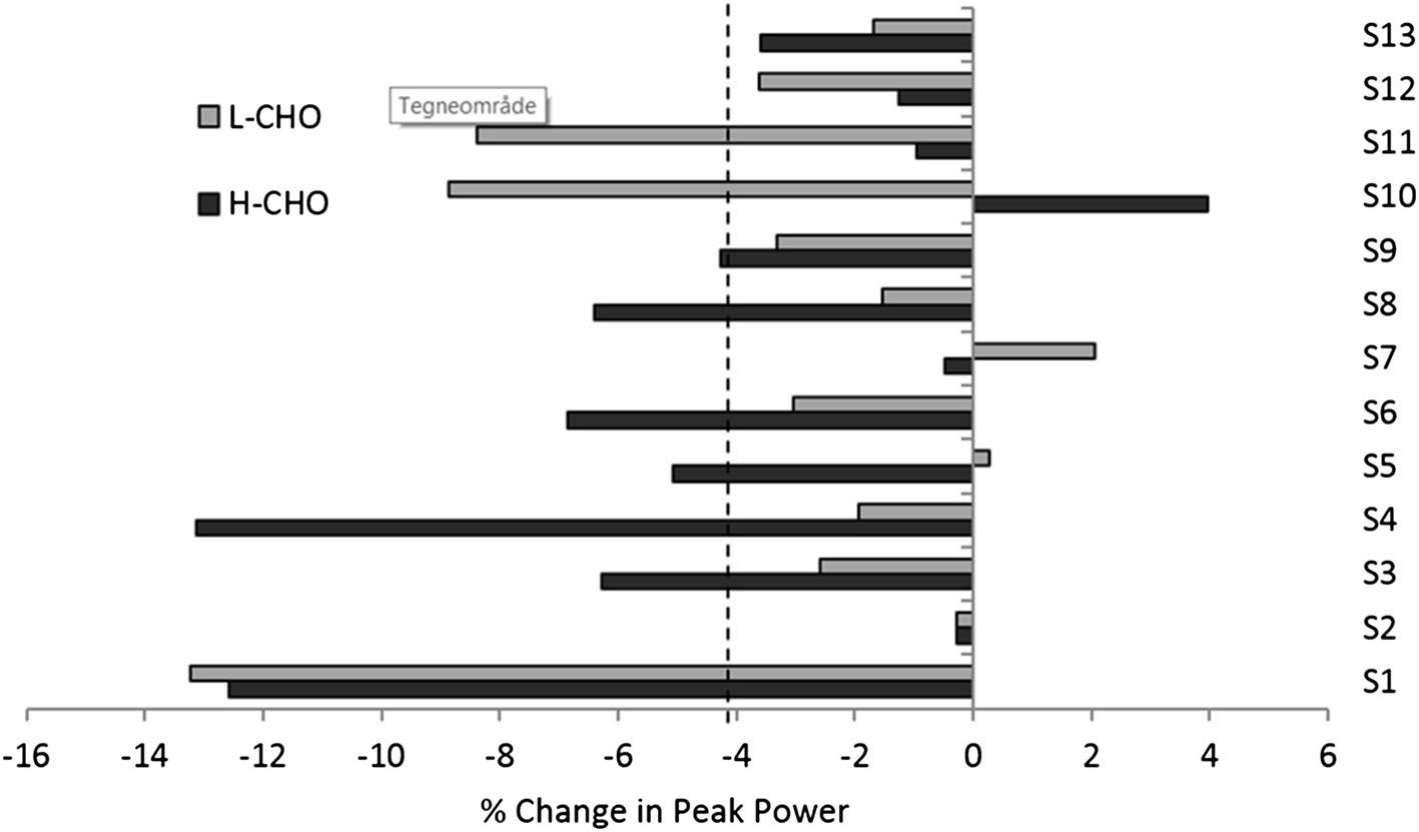
Percentage change in peak power achieved during the incremental, maximal exercise test for each participant following the intensified training period with L-CHO and H-CHO. *Dashed line* indicates significant group mean change (*P* < 0.01)

There was no change in $\dot{V}O_{2\hbox{max} }$ following IT. However, in both L-CHO and H-CHO trials, IT resulted in decreased peak power in the maximal incremental exercise test compared to baseline (375 ± 37 vs. 391 ± 37 W, main effect of IT *P* = 0.001) (Fig. 2), reduced maximal heart rate (179 ± 8 vs. 190 ± 10 bpm, main effect of IT *P* < 0.001), reduced haematocrit (39 ± 2 vs. 42 ± 2 %, main effect of IT *P* < 0.001) and a 14 ± 10 % increase in plasma volume at rest (PV) (main effect of IT *P* < 0.001) (Table 3), with no significant differences between trials.

**Table 3:** Haematological variables and plasma volume changes before and after acute maximal exercise, and before and after the 8-day intensified training intervention

	8 days intensified training			
	Pre IT		Post IT	
	Pre-exercise	Post-exercise	Pre-exercise	Post-exercise
RBC (×10^9^/L)				
H-CHO	4.60 ± 0.22	4.87 ± 0.28*	4.25 ± 0.19^†^	4.47 ± 0.23^†^*
L-CHO	4.60 ± 0.16	4.87 ± 0.16*	4.29 ± 0.23^†^	4.52 ± 0.23^†^*
Hb (g/dL)				
H-CHO	14.3 ± 0.8	15.1 ± 0.9*	13.2 ± 0.8^†^	13.9 ± 0.9^†^*
L-CHO	14.1 ± 0.8	14.9 ± 0.9*	13.0 ± 0.8^†^	13.6 ± 0.9^†^*
HCT (%)				
H-CHO	41.9 ± 2.4	45.0 ± 2.7*	39.0 ± 1.7^†^	41.3 ± 1.8^†^*
L-CHO	42.0 ± 1.3	45.1 ± 1.7*	39.2 ± 2.0^†^	41.7 ± 2.0^†^*
PV (% of baseline)				
H-CHO	100 ± 0	89 ± 4*	114 ± 11^†^	104 ± 12^†^*
L-CHO	100 ± 0	90 ± 5*	114 ± 10^†^	104 ± 9^†^*

Data are mean ± SD

*RBC* red blood cell count, *PV* plasma volume, *Hb* haemoglobin, *HCT* haematocrit

* Significant effect of acute maximal exercise (P < 0.001), ^†^ significantly different from the same time point pre-intensified training (*P* < 0.001)

**Fig. 3:**
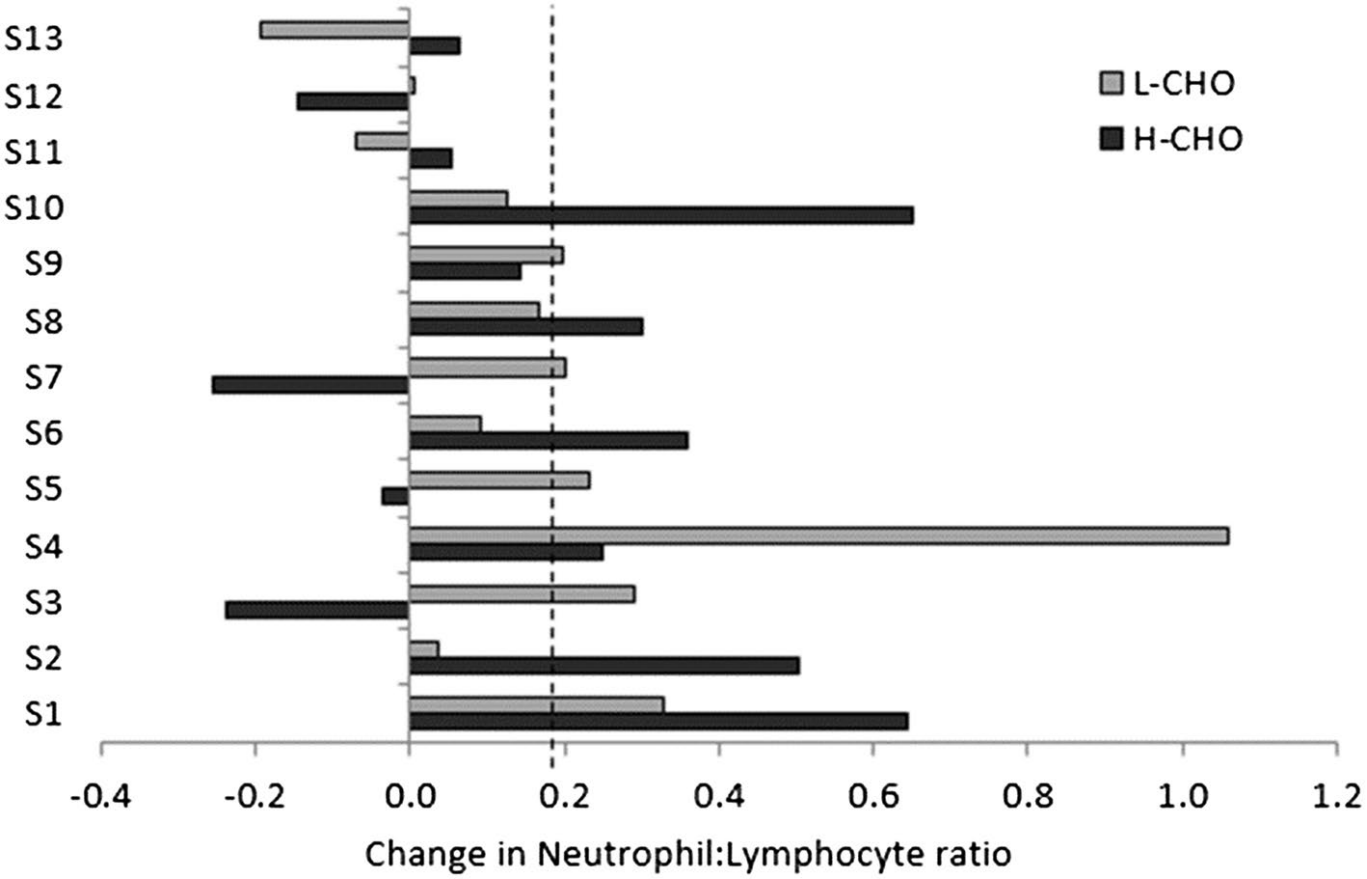
Change in neutrophil:lymphocyte ratio for each participant following intensified training with L-CHO and H-CHO. *Dashed line* indicates significant group mean change (*P* < 0.05)

Circulating total leukocytes and neutrophils at rest were increased with IT (*P* < 0.01, Table 4), as was the percentage of lymphocytes that were CD4+ (29 ± 6 vs. 26 ± 7 %, main effect of IT *P* < 0.05), with no difference between trials. Seven of the 13 participants had somewhat lower than normal numbers of CD4+ cells (<0.5 × 10^9^ cells and <30 % of total lymphocytes) at baseline. The exercise-induced increase in CD4+ cells was significantly reduced following IT (0.10 ± 0.14 × 10^9^ vs. 0.19 ± 0.11 × 10^9^, main effect of IT *P* < 0.01) with no significant difference between trials. There were no significant changes in the numbers of CD24+ CD25+ CD127^low/−^Treg cells following intensified training in either trial. Resting neutrophil:lymphocyte ratio was significantly increased following IT (Fig. 3, main effect of IT *P* < 0.05) with no significant difference between trials.

**Table 4:** Leukocyte counts before and after acute maximal exercise, and before and after 8 days of intensified training in L-CHO and H-CHO trials

	8 days intensified training			
	Pre-training		Post-training	
	Pre-exercise	Post-exercise	Pre-exercise	Post-exercise
WBC				
HCHO	5.30 ± 0.85	8.34 ± 1.49*	5.99 ± 1.00^†^	9.14 ± 1.70 *^†^
LCHO	5.43 ± 0.93	7.93 ± 1.27*	5.92 ± 0.94^†^	8.31 ± 1.17 *^†^
NEUT				
HCHO	2.45 ± 0.49	3.17 ± 0.75*	2.93 ± 0.78^†^	3.99 ± 1.08 *^†^
LCHO	2.58 ± 0.69	3.26 ± 0.77*	3.00 ± 0.78^†^	3.74 ± 0.81*^†^
MONO				
HCHO	0.51 ± 0.12	0.75 ± 0.22*	0.59 ± 0.15	0.89 ± 0.33*
LCHO	0.53 ± 0.13	0.72 ± 0.18*	0.55 ± 0.14	0.75 ± 0.21*
LYMPH				
HCHO	2.17 ± 0.61	4.08 ± 1.07*^#^	2.26 ± 0.65	4.03 ± 0.98*^#^
LCHO	2.12 ± 0.47	3.65 ± 0.66*	2.17 ± 0.47	3.53 ± 0.95*
CD4^+^				
HCHO	0.55 ± 0.21	0.77 ± 0.23*^#^	0.58 ± 0.20	0.71 ± 0.20*†^#^
LCHO	0.54 ± 0.20	0.69 ± 0.23*	0.58 ± 0.20	0.65 ± 0.18*†
CD4+ CD25+				
HCHO	0.14 ± 0.04	0.22 ± 0.07*	0.14 ± 0.05	0.19 ± 0.05*
LCHO	0.14 ± 0.05	0.18 ± 0.06*	0.13 ± 0.04	0.16 ± 0.05*
CD4+ CD25+ CD127^low/^−				
HCHO	0.05 ± 0.02	0.06 ± 0.03*	0.05 ± 0.02	0.06 ± 0.02*
LCHO	0.05 ± 0.02	0.06 ± 0.02*	0.05 ± 0.02	0.05 ± 0.02*

Data are mean ± SD. Cell counts are corrected for changes in plasma volume

*WBC* total white blood cells, *NEUT* neutrophils, *MONO* monocytes, *LYMPH* lymphocytes, *CD4*+ lymphocytes expressing CD4 (T helper), *CD4* *+* *CD25* *+* lymphocytes expressing CD4 and CD25, *CD4*+ *CD25*+ *CD127* ^*low/*−^lymphocytes expressing CD4 and CD25, but not CD127 (T regulatory)

* Significant effect of acute maximal exercise (*P* < 0.01), ^†^ significant difference from the same time point pre-intensified training (*P* < 0.01), ^#^ significant difference from L-CHO at the same time point (*P* < 0.05)

**Fig. 4:**
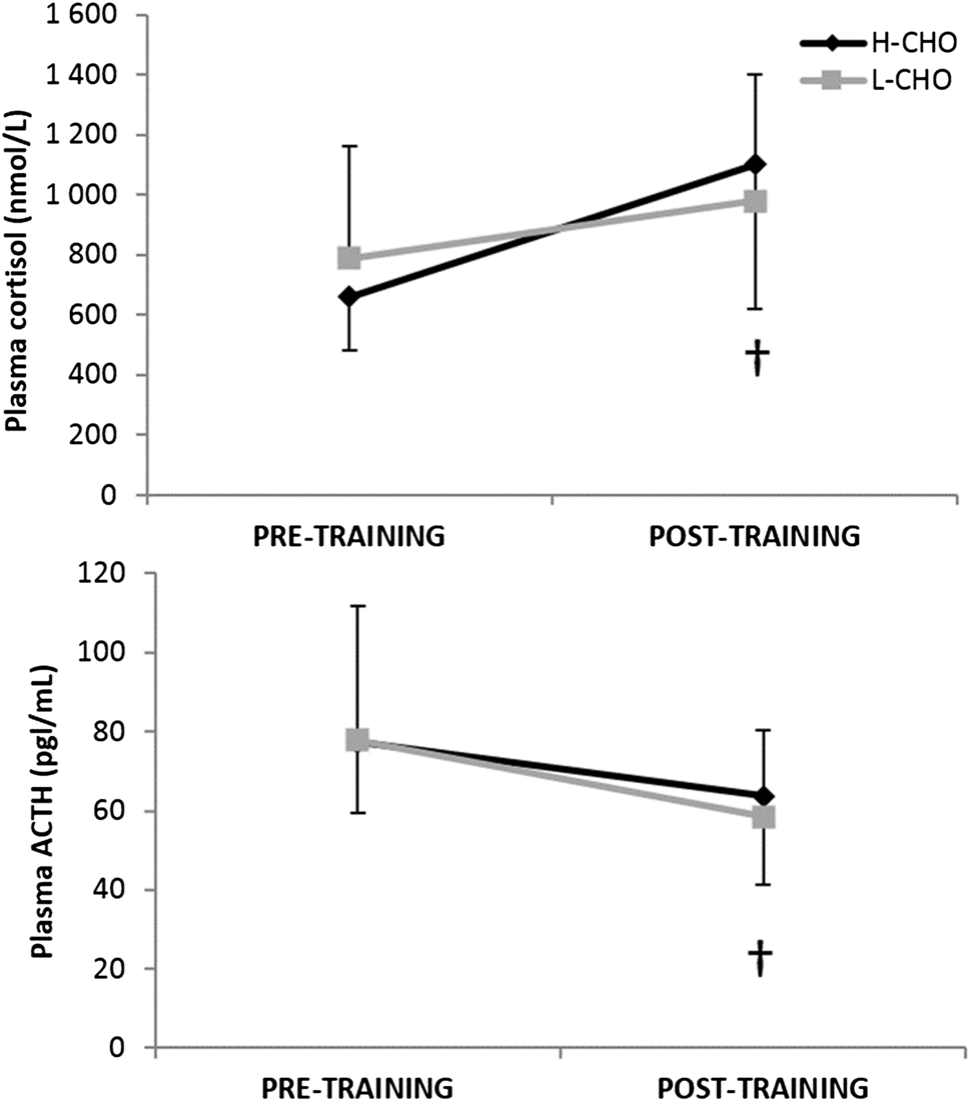
Resting plasma cortisol (**a**) and ACTH (**b**) following intensified training. Values are mean ± 95 % CI. ^†^Significant difference from pre-intensified training, *P* < 0.05. Corrected for change in plasma volume

**Fig. 5:**
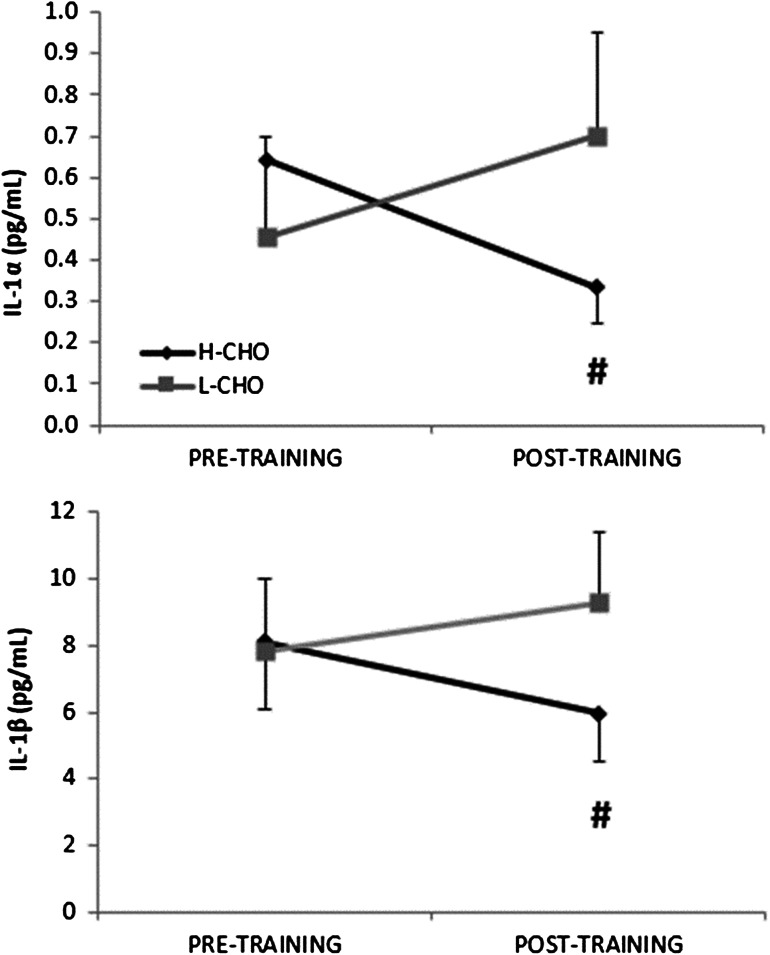
Resting antigen-stimulated production of interleukin-1α (**a**) and interleukin-1β (**b**) by whole blood culture before and after intensified training with L-CHO and H-CHO. Values are mean ± 95 % CI. ^#^Significant difference between L-CHO and H-CHO (*P* < 0.05)

Resting plasma ACTH was lower following IT (61 (95 % CI 46–82) pg/mL vs. 78 (57–107) pg/mL, main effect of IT *P* < 0.01), while resting plasma cortisol concentration was increased (1037 (95 % CI 648–1661) nmol/L vs. 722 (508–1025) nmol/L, main effect of IT *P* < 0.05) (Fig. 4). There was no significant effect of IT on the exercise-induced change in ACTH or cortisol, nor were there any significant differences between L-CHO and H-CHO in either hormone. Following IT, the production of IL-1α at rest in response to antigen challenge was higher in L-CHO compared to H-CHO (0.70 (95 % CI 0.52–0.95) pg/mL vs. 0.33 (0.24–0.45) pg/ml, interaction effect *P* < 0.01), as was production of IL-1β (9.3 (95 % CI 7–10.4) pg/mL vs. 6.0 (5.0–7.8) pg/ml, interaction effect *P* < 0.05) (Fig. 5). Production of TNFα in response to antigen challenge also tended to be higher in L-CHO post-IT (*P* = 0.063). Antigen-stimulated production of other measured cytokines (IL-2, IL-4, IL-6, IL-8, IFN-γ) was not significantly altered by IT and did not differ between trials. All of the measured haematological and immunological variables were significantly increased immediately following acute maximal exercise (main effect of acute exercise *P* < 0.05) with no difference between trials.

**Fig. 6:**
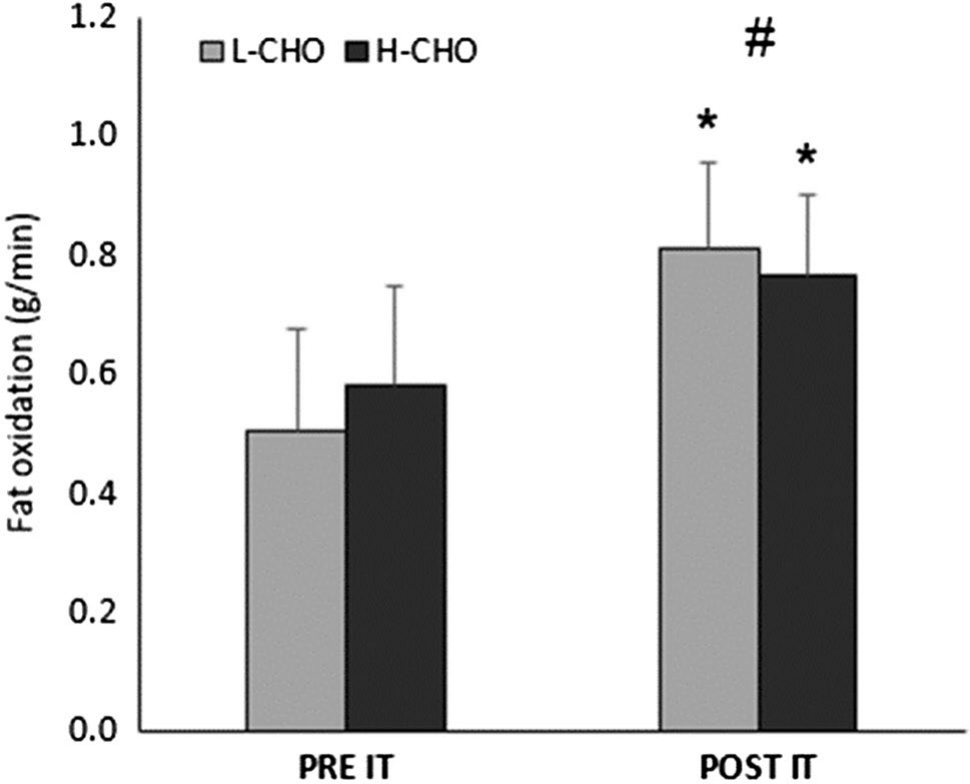
Average fat oxidation during the first five stages (60–235 W) of the incremental test before and after intensified training in L-CHO and H-CHO trials. Values are mean + SD. *Significant difference from pre-intensified training in both trials, *P* < 0.01. ^#^Significant interaction effect, *P* < 0.05

Submaximal fat oxidation during the first five stages of the incremental test was significantly increased following IT (Fig. 6, main effect of IT *P* < 0.01), with this increase being greater in L-CHO (L-CHO: 0.51 ± 0.17 pre IT to 0.81 ± 0.14 g/min post-IT vs. H-CHO: 0.58 ± 0.17 pre-IT to 0.77 ± 0.14 g/min post-IT, interaction effect *P* < 0.05). The workload at which maximal fat oxidation occurred was also increased following IT (226 ± 45 vs. 181 ± 41 W, main effect of IT *P* < 0.01) with no difference between trials.

## Discussion

The main finding of the current study is that an 8-day period of IT in highly trained cyclists induces some immunological, haematological, physiological and exercise-related changes consistent with the symptoms of overreaching. However, carbohydrate supplementation in and around the exercise training occasion, in addition to a high carbohydrate-containing diet (>7 g CHO/kg/day), did not appear to reduce the associated maximal power output decrement or ameliorate hormonal and immunological disturbances. This is in agreement with Snyder et al. (1995), who found that even when muscle glycogen was maintained via carbohydrate supplementation, competitive cyclists still displayed symptoms of overtraining following 15 days of IT. In contrast, Achten et al. (2004) found that an increase in dietary carbohydrate from 5.4 to 8.5 g CHO/kg/day better maintained physical performance in runners undergoing a period of IT, reducing the symptoms of overreaching. Similarly, Costa et al. (2005) found that consumption of a very high carbohydrate diet (12 g CHO/kg/day) during IT attenuated cortisol release and increased salivary secretory immunoglobulin-A concentration compared to a self-selected, and more moderate, CHO intake of 5.9 g/kg/day. Halson et al. (2004) found that ingestion of a high carbohydrate beverage before, during and after training sessions over the course of an 8-day period of intensified cycling training better maintained performance and reduced symptoms of overreaching compared to a low carbohydrate placebo (9.4 vs. 6.4 g/kg/day). However, in the current study, total daily carbohydrate intake in the control group was higher than in these studies at 7.2 g/kg/day, a daily amount classed as ‘high’ in the International Olympic Committee’s latest consensus on sports nutrition (Burke et al. 2011). It is possible that this intake was already sufficient to provide some protection against immunological disturbances and other symptoms of overreaching. Another possible explanation is differences in training during the IT period. Compared with the study of Halson et al. (2004), participants in the current study completed a greater total volume of cycling (23.5 vs. 15.3 h/week), but less high-intensity training (6.5 vs. 10.5 h/week). Since muscle glycogen utilization is related to exercise intensity (Saltin and Karlsson 1971), it is possible to speculate that the lower number of hours of high-intensity training in the current study may have resulted in lower glycogen depletion, and that CHO supplementation was therefore less critical in maintaining performance. In addition, participants in the current study were highly trained, with greater aerobic capacity than in previous studies, and may therefore have been more resilient to detrimental effects of the IT period. There was high inter-individual variation in the magnitude of the performance change following the IT period, with this ranging from −13 to +4 % across both trials. As such, for some of these well-trained athletes, the prescribed training load does not appear to have been sufficient to induce a true state of overreaching. The fact that some individuals appear better able to tolerate IT periods than others is an important consideration for coaches and practitioners, and highlights the need for individualised training programmes. The IT resulted in a marked reduction in resting haematocrit, haemoglobin and red blood cell concentration, likely primarily indicative of a pronounced plasma volume expansion. It is well reported that physical training stimulates an increase in plasma volume (Convertino 1991) and it is likely that the changes observed in the current study are primarily indicative of adaptation rather than overreaching. However, it is also possible that these changes were due, at least in part, to changes in red cell destruction or production as a result of IT. Indeed, strenuous endurance exercise has been associated with increased intravascular haemolysis, due to oxidative damage to red blood cells (Smith 1995).

Whether or not the changes in immune variables observed in the current study following IT might make athletes more susceptible to URTI is not clear. The ~315 nmol/L increase in resting plasma cortisol may have negative implications for host defence, particularly since mean concentration at baseline was already towards the upper end of the normal range. Several studies have investigated the effect of IT on plasma cortisol concentrations, with the majority reporting no significant change in resting plasma cortisol following IT (Hooper et al. 1993; Mackinnon et al. 1997; Urhausen et al. 1998). It is possible that the increase observed in the current study, along with elevations in circulating leukocytes, neutrophils, CD4+ lymphocytes and neutrophil:lymphocyte ratio, may be only a transient effect caused by incomplete recovery from the previous exercise bout ~13 h earlier.

Handzlik et al. (2013) recently reported that endurance trained athletes with a high training load (~15 h/wk) had a significantly higher proportion of regulatory T cells as a percentage of total lymphocytes and higher antigen-stimulated in vitro IL-10 production compared with sedentary individuals. However, in the current study, there was no significant effect of IT on absolute or relative numbers of Treg cells or antigen-stimulated IL-10 production, suggesting that these variables are not altered by short-term increases in training load in individuals already training ~9 h/wk. However, antigen-stimulated production of both IL-1α and IL-1β was both reduced following IT in H-CHO and increased following IT in L-CHO. Type 1 cytokines, such as IL-1, promote cellular immunity by stimulating the functional activity of T cytotoxic cells, natural killer cells and activated macrophages (Gleeson et al. 2013). Reduced production of these cytokines in response to an immune challenge may therefore have negative implications for host defence. Similar to the results observed for the H-CHO trial, Morgado et al. (2012) reported impaired production of IL-1β from dendritic cells and monocytes in swimmers following IT. However, that IL-1α and IL-1 β production should be increased following IT in L-CHO appears counterintuitive, and further research is necessary to corroborate these findings. Nevertheless, it is clear that increasing carbohydrate intake during exercise, from 20 to 60 g/h, does not provide any additional benefit in preserving the measured immune parameters during IT in this cohort of highly trained cyclists. In fact, since neither maximal power output nor immunity was better maintained with a high carbohydrate intake, and increases in fat oxidation were lower, it could be argued that ingesting a moderate (~20 g/h) carbohydrate dose during short-term IT may be more advantageous than a higher carbohydrate dose, at least when athletes already have a high daily intake of dietary carbohydrate (>7 g CHO/kg/day). This intake during exercise appears sufficient to help prevent large maximal power output decrements and preserve immunity, while facilitating greater adaptation in terms of fat oxidation. Whether this improved capacity to oxidize fat has subsequent performance benefits once recovery from IT is complete is unknown.

The mean maximal oxygen uptake of participants in the current study was 72.2 mL/kg/min, and to our knowledge, no previous study has attempted to intentionally overreach such highly endurance trained athletes. The current data indicate that these athletes are relatively robust to increases in training load and can tolerate multiple consecutive days of IT without large reductions in maximal power output or severe immune disturbances. However, since many of the participants had lower than expected numbers of CD4+ cells and higher than expected plasma cortisol already at baseline, it is possible that their habitually high training loads may already be associated with some mild immunodepression.

The fact, that participants consumed an otherwise self-selected diet may be considered a limitation of the current study. However, previous studies have already established that increasing total daily carbohydrate intake can alleviate the symptoms of overreaching (Achten et al. 2004; Costa et al. 2005). In contrast, we wished to determine whether or not it is possible to preserve performance and immunity during intensified training merely by manipulating carbohydrate intake in and around training sessions in athletes otherwise consuming a “normal” moderate–high carbohydrate diet.

It should be noted that, since the intervention beverages were not isocaloric, trials differed not only in carbohydrate intake but also in total energy intake, as well as in the protein content of the post-exercise recovery drinks. However, within the range tested in the current study, neither of these appears to influence the responses to IT. The lack of a significant difference in weight change between trials, despite a difference in energy intake, may indicate limited validity of food diaries. Alternatively, it is possible to speculate that participants may have felt more tired and therefore reduced their general physical activity levels when on the L-CHO trial, despite no differences observed between trials in work done during the training sessions themselves.

## Conclusion

Eight days of IT results in a number of changes consistent with the symptoms of short-term overreaching in highly trained cyclists, although both immunological and physiological changes are relatively modest. When ingesting an otherwise self-selected diet, a high carbohydrate intake immediately before, during and after training sessions was not able to better maintain maximal power output, or alleviate physiological/immunological disturbances compared to a moderate carbohydrate intake. The results also highlight the importance of considering plasma volume changes when interpreting results from training studies, since even highly trained individuals demonstrate a pronounced plasma volume expansion following just 8 days of IT.

## Abbreviation

ACSM: American College of Sports Medicine
ACTH: Adrenocorticotropic hormone
H-CHO: High carbohydrate-supplemented condition
HR_max_: Maximal heart rate
IFN-γ: Interferon gamma
IL: Interleukin
IT: Intensified training
L-CHO: Low carbohydrate-supplemented condition
PBS: Phosphate-buffered saline
TNF-α: Tumour necrosis factor alpha
Treg: Regulatory T cell
URTI: Upper respiratory tract infection
$\dot{V}CO_{2}$: Carbon dioxide production
$$\dot{V}E$$: Minute ventilation
$$\dot{V}O_{2}$$: Oxygen uptake
$\dot{V}O_{2\hbox{max} }$: Maximal oxygen uptake
